# Citrus Postharvest Green Mold: Recent Advances in Fungal Pathogenicity and Fruit Resistance

**DOI:** 10.3390/microorganisms8030449

**Published:** 2020-03-23

**Authors:** Yulin Cheng, Yunlong Lin, Haohao Cao, Zhengguo Li

**Affiliations:** 1 Key Laboratory of Plant Hormones and Development Regulation of Chongqing, School of Life Sciences, Chongqing University, Chongqing 401331, Chinahaohaocao@126.com (H.C.); 2 Center of Plant Functional Genomics, Institute of Advanced Interdisciplinary Studies, Chongqing University, Chongqing 401331, China

**Keywords:** citrus fruit, postharvest disease, *Penicillium digitatum*, pathogenesis, disease resistance

## Abstract

As the major postharvest disease of citrus fruit, postharvest green mold is caused by the necrotrophic fungus *Penicillium digitatum* (*Pd*), which leads to huge economic losses worldwide. Fungicides are still the main method currently used to control postharvest green mold in citrus fruit storage. Investigating molecular mechanisms of plant–pathogen interactions, including pathogenicity and plant resistance, is crucial for developing novel and safer strategies for effectively controlling plant diseases. Despite fruit–pathogen interactions remaining relatively unexplored compared with well-studied leaf–pathogen interactions, progress has occurred in the citrus fruit–*Pd* interaction in recent years, mainly due to their genome sequencing and establishment or optimization of their genetic transformation systems. Recent advances in *Pd* pathogenicity on citrus fruit and fruit resistance against *Pd* infection are summarized in this review.

## 1. Introduction

Citrus is an important fruit crop worldwide, especially in tropical and subtropical regions around the world, and citrus fruit contains many nutritional components beneficial to human health [1]. During the postharvest stage, including handling, shipping, storing, and marketing, fruit is subjected to a series of biotic or abiotic stresses, and fruit decay and risks to food safety caused by postharvest fungal diseases are some of the most serious problems [2]. Green mold and blue mold, caused by *Penicillium digitatum* (*Pd*) and *Penicillium italicum* (*PI*), respectively, are the two most important postharvest diseases in all citrus production areas [3]. Postharvest green mold, which is the main factor resulting in citrus fruit decay, leads to huge economic losses worldwide every year and can account for up to 90% of the total citrus postharvest losses, especially in arid zones and subtropical climates [4,5]. As the current control method used for other postharvest fungal diseases on fruit, the mass application of fungicides is still the main control of citrus postharvest green mold in citrus fruit storage [2,6]. The use of synthetic fungicides has been regarded as the standard procedure for controlling citrus postharvest green mold in many citrus-producing areas for decades [7]. However, fungicide resistance and noticeable health or environmental risks derived from synthetic fungicides are increasingly concerning [6,8]. Thus, developing rational alternatives for controlling citrus postharvest green mold is an imperative.

Plant–pathogen interactions, mainly including pathogenicity and plant resistance, are the central issue in plant pathology research because investigating how plants and pathogens recognize each other and differentiate to establish either a successful or an unsuccessful relationship is crucial in this field of investigation [9]. Many studies have been performed to investigate plant–pathogen interactions, indicating that plant–pathogen interactions are complex [10,11]. By studying the pathogenicity of plant pathogens, two innovative strategies—host-induced gene silencing (HIGS), which is an RNA interference strategy and involves host expression of double-stranded RNA (dsRNA) targeting essential pathogen genes, and spray-induced gene silencing (SIGS), which involves inhibition of plant pathogens through a direct spray of dsRNA targeting essential pathogen genes—have been used to control plant diseases caused by fungal and oomycete pathogens [12]. HIGS of *Verticillium dahliae VdH1* in transgenic cotton plants conferred effective resistance to the wilt disease pathogen *V. dahliae* [13], and spraying of long dsRNAs targeting three fungal cytochrome P450 lanosterol C-14α-demethylases significantly improved barley resistance to necrotrophic fungus *Fusarium graminearum* [14]. By studying plant resistance to pathogens, transgenesis by overexpressing disease resistance genes (R genes) or positive regulators of plant defense and silencing or genome editing of disease susceptibility genes (S genes) has been used to control different plant diseases caused by fungi, bacteria, and viruses [15,16]. The revolutionary biotechnology known as genome editing will probably become a standard tool in plant breeding of disease resistant crops in the future [17]. Genome editing of the conserved S gene *MLO* in different crops, including wheat and tomato, conferred heritable resistance to powdery mildew [18,19]. Thus, investigating the molecular mechanisms of plant–pathogen interactions, including pathogenicity and plant resistance, is crucial for developing novel and safer strategies for effectively controlling plant diseases.

Pathogens can infect different plant tissues and the leaf is a major tissue infected by pathogens [20]. Knowledge of plant–pathogen interactions has mainly been obtained from leaf diseases [11,21], and fruit–pathogen interactions remain relatively unexplored compared with the well-studied leaf–pathogen interactions [2]. Nevertheless, the study of fruit–pathogen interactions has gained increased interest and progress has occurred in the understanding of fungal pathogenicity and fruit resistance [2]. In the citrus fruit–*Pd* interaction, *Pd* genome sequencing and establishment of *Pd* genetic transformation system have been completed recently [5,22,23], which have greatly promoted the understanding of *Pd* pathogenicity at molecular levels [24]. The recent genome sequencing of citrus, optimization of the citrus genetic transformation system, and establishment of citrus CRISPR/Cas9 gene editing system [25,26,27,28] have promoted the study of citrus disease resistance [26,29]. 

In this review, we mainly introduce recent advances in fungal pathogenicity and fruit resistance of citrus postharvest green mold, which provide significant insights into fruit–pathogen interactions and is beneficial for developing novel and safer strategies for controlling citrus postharvest green mold. 

## 2. Infection Characteristics of *Pd* on Citrus Fruit

### 2.1. Infection Process

As a necrotrophic fungal pathogen, *Pd* infects citrus fruit through a variety of wounds originating from mechanical damage and environmental factors including cold, burn, wind, hail, and insects [30]. *Pd* conidia dispersed by wind or raindrops onto the surface of citrus fruit can germinate to produce germ tubes under suitable conditions. After penetrating into pericarp cells by germ tubes, *Pd* extends into mesocarp cells and gradually invades the adjacent cells [31]. In the later infection process, white mycelia and newly generated grayish conidia are produced on infected citrus fruit, which is considered the typical disease symptom of citrus postharvest green mold [31,32]. Upon *Pd* infection, citrus fruit initially shows a water logging symptom and is finally rotted because infected pericarp cells and mesocarp cells are plasmolyzed and their inclusions and organelles are coagulated, dark, and digested [31]. 

### 2.2. Influence Factors of Pd Spore Germination

The germination of conidia is a key step of *Pd* infection on citrus fruit and some factors influencing *Pd* spore germination have been reported. These *Pd* conidia around the wounds of citrus fruit germinate very well but other *Pd* conidia far from these wounds rarely germinate [31], which is probably due to the signal stimulation of spore germination from wounds [33]. The volatiles, including limonene, myrcene, α-pinene, and β-pinene, emitted from the ruptured oil glands in wounded peel tissues were proven to promote the germination of *Pd* conidia [34]. The constituents of orange juice, mainly including sugars and organic acids, also stimulate the germination of *Pd* conidia [35]. 

The effect of water activity (a_w_) and temperature on the germination of *Pd* conidia was also investigated [36,37]. *Pd* conidia are able to germinate in the temperature range of 4–30 °C and the optimum temperature for the germination of *Pd* conidia is 25 °C [36]. In addition, *Pd* conidia are able to germinate in the a_w_ range 0.90–1.00, and no germination is observed under 0.90 a_w_ [36,37].

### 2.3. Mycotoxin during Pd Infection

Mycotoxin is a severe problem for public health and the analysis of potential mycotoxins in fruits infected by postharvest fungal pathogens are important for their quality control and safety [38]. Thermogenic alkaloids, including tryptoquialanine A and tryptoquialanine C, were detected in citrus fruit infected by *Pd* [38,39,40]. However, no other mycotoxins have been reported in *Pd* infection on citrus fruit. The genome sequencing of *Pd* indicates the absence of the biosynthesis of patulin, which is a mycotoxin in *Penicillium expansum*, another postharvest fungal pathogen closely related to *Pd* [5]. Clusters of genes (such as *gsf*, *avf*, and *cyp*) responsible for the synthesis of important mycotoxins in other fungal species, including griseofulvin, viridicatumtoxin, fumonisin, clavines, aflatoxins, sterigmatocystin, citrinin, ergot alkaloid, lovastatin, and paxilline, are also absent in the *Pd* genome [5], which can explain why these mycotoxins have not been reported in *Pd* infection on citrus fruit. 

As other secondary metabolites, many mycotoxins play important roles in fungal pathogenicity [41,42]. The mycotoxin patulin is involved in the pathogenicity of another postharvest fungal pathogen *P. expansum* [43], which implies the putative role of tryptoquialanines in *Pd* pathogenicity. Deleting a tryptoquialanine-production-related gene *tqaA* in *Pd* resulted in complete loss of tryptoquialanine production but no significant difference was observed in *Pd* virulence between the Δ*tqaA* mutant and the wild type strain, suggesting that tryptoquialanines are not involved in *Pd* pathogenicity [44]. Noticeably, tryptoquialanines may be involved in the protection of decayed citrus from insects because tryptoquialanine A shows high insecticide activity for *Aedes Aegypti* [39]. 

## 3. Pathogenic Mechanisms of *Pd*

**Figure 1:**
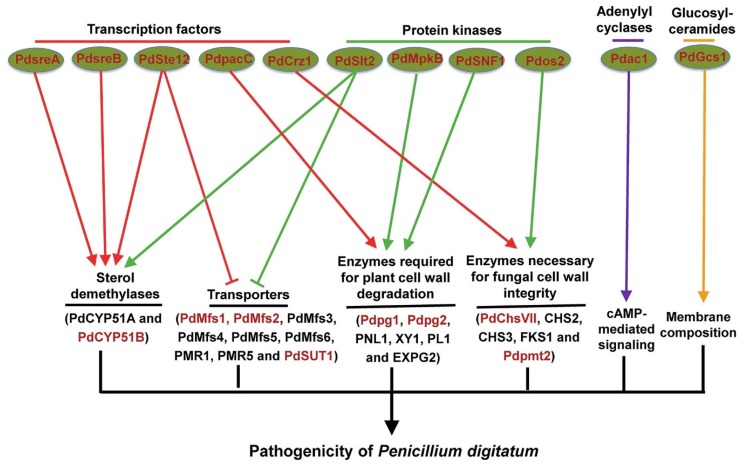
Overview of identified genes (red-colored) involved in the pathogenicity of *Penicillium digitatum*.

The achievement of *Pd* complete genome, which is the first genome sequencing of a phytopathogenic *Penicillium* species [5], provides an important basis for understanding the pathogenic mechanisms of *Pd*. Genome sequencing shows that *Pd* has a much smaller gene content (around 26 Mb), consistent with a more specialized lifestyle, compared with the closely-related but nonphytopathogenic *P. chrysogenum* [5]. Whole genome variation analysis revealed very high similarity among globally distributed *Pd* isolates, which points to a recent and global expansion of a single lineage in *Pd* [45]. In addition, putative *Pd* pathogenicity-related genes were identified using a subtractive complementary DNA (cDNA) library of *Pd*-infected citrus peel tissues and comparison against the available *Pd* genome sequences [7]. Noticeably, a total of 19 important pathogenicity factors that are responsible for *Pd* virulence on citrus fruit and mainly encode transcription factors, cell wall-related enzymes, protein kinases, and transporters (Figure 1) have been identified using *Agrobacterium tumefaciens*-mediated transformation system of *Pd* [22,23]. 

### 3.1. Transcription Factors in Pd Pathogenicity

Transcription factors (TFs)-mediated transcriptional regulation plays important roles in fungal pathogenicity, and many pathogenicity-related transcription factors have been reported in a number of plant fungal pathogens [46]. As the fungus-specific transcription factor, Ste12 is the downstream target of the conserved mitogen-activated protein kinase (MAPK) pathway and is required for fungal pathogenicity in different plant pathogenic fungi [47]. The role of Ste12 in *Pd* pathogenicity was also investigated and the *ΔPdSte12* mutant exhibited reduced fungal growth and disease symptoms on citrus fruit [48,49]. Further gene expression analysis revealed that several *Pd* genes involved in transport including two ABC transporters (PMR1 and PMR5), six MFS transporters (PdMfs1-6), and a putative sucrose transporter PdSUT1 were up regulated in the Δ*PdSte12* mutant compared with the wild type strain [48]. Among these transporters, PdMfs1, PdMfs2, and PdSUT1 were reported to be required for *Pd* pathogenicity (see Section 3.4). Two sterol demethylase genes, including *CYP51* (also known as *PdCYP51A*) and *PdCYP51B*, which are essential for ergosterol synthesis in fungal membranes and are known for their involvement in *Pd* resistance to imazalil and other fungicides inhibiting ergosterol synthesis [50], were downregulated in the Δ*PdSte12* mutant [48]. A recent study showed that PdCYP51B also plays a role in *Pd* pathogenicity (see Section 3.5). Taken together, transcription factor PdSte12 is involved in *Pd* pathogenicity possibly via negative regulation of transporter genes and positive regulation of sterol demethylase genes. 

Transcription factor PacC belongs to the conserved PacC/Rim101 signaling cascade to modulate pH in infection of plant pathogenic fungi [51]. Noticeable pH differences exist between healthy oranges and *Pd*-infected oranges [52], and disruption of *PdpacC* resulted in attenuated *Pd* virulence on citrus fruit [53], which indicates that PacC-mediated pH modulation is also involved in *Pd* pathogenicity. Further gene expression analysis showed that two cell wall degrading enzyme genes, including the polygalacturonase *Pdpg2* and the pectin lyase *Pdpnl1* (also known as PNL1), were upregulated in the wild type strain but not or weakly upregulated in the Δ*PdpacC* mutant upon *Pd* infection [53]. Noticeably, *Pdpg2* was also reported to be required for *Pd* pathogenicity (see Section 3.2). These results indicate that PacC-mediated pH modulation of citrus fruit promotes *Pd* infection probably by leading to an optimal pH for specific cell wall degrading enzymes to degrade citrus peel. 

The Ca^2+^/calmodulin-dependent pathway plays an important role in fungal pathogenicity [54], and the calcineurin-responsive transcription factor Crz1 is required for virulence of different fungal pathogens, including *Botrytis cinerea*, *Magnaporthe oryzae*, and *Aspergillus fumigatus* [55,56,57]. The role of Crz1 in *Pd* pathogenicity was investigated and the Δ*PdCrz1* mutant was found to be defective in *Pd* virulence on citrus fruit [58]. Cell wall integrity was defective and three cell wall synthase genes (*CHS2*, *CHS3*, and *FKS1*) were significantly downregulated in the Δ*PdCrz1* mutant compared with the wild type strain [58]. These results indicate that the calcineurin-responsive transcription factor Crz1 is involved in *Pd* pathogenicity probably via positive regulation of cell wall synthase genes to maintain the cell wall integrity of *Pd*. 

Sterol regulatory element binding proteins (SREBPs) are a family of transcription factors that regulate sterol homeostasis in many eukaryotes [59]. SREBP transcription factors play a key role in the regulation for ergosterol biosynthesis in a number of fungal species, and are also required for virulence of several human fungal pathogens including *Cryptococcus neoformans* and *A. fumigatus* [60]. However, little is known about the role of SREBP transcription factors in plant fungal pathogens. The putative roles of two SREBP transcription factor encoding genes (*PdsreA* and *PdsreB*) in *Pd* pathogenicity were investigated and all mutants including Δ*PdsreA*, Δ*PdsreB*, and the double mutant showed reduced ergosterol contents and virulence on citrus fruit [61,62]. In addition, the sterol demethylase gene *PdCYP51B*, which plays a role in *Pd* pathogenicity (see Section 3.5) was downregulated in Δ*PdsreA* and Δ*PdsreB* mutants compared with the wild type strain [61,62]. These results indicate that SREBP transcription factors are required for *Pd* pathogenicity possibly via positive regulation of sterol demethylase genes.

### 3.2. Cell Wall-Related Enzymes in Pd Pathogenicity

Cell wall-related enzymes play an important role in plant–pathogen interactions because fungal pathogens invade host tissues by degrading the plant cell wall to obtain nutrients, and cell wall integrity of fungal pathogens is important for protection from host degradation [63,64]. Many *Pd* genes encoding cell wall-related enzymes, of which cell wall-degrading enzymes are predominant, are significantly upregulated during *Pd* infection on citrus fruit [7], which suggests that cell wall-related enzymes may be involved in *Pd* pathogenicity. Carbohydrate-active enzymes (CAZYmes) are proven to regulate fungal pathogenicity probably by degrading plant cell wall in different phytopathogenic fungi [65]. The role of two major polygalacturonases (PGs), PG1 and PG2, which belong to CAZYmes, in *Pd* pathogenicity was investigated [66]. Both Δ*Pdpg1* and Δ*Pdpg2* mutants showed reduced disease symptoms on citrus fruit compared with the wild type strain but the defects in the development of green mold and the corresponding galacturonic acid production and tissue softening were more obvious in the Δ*Pdpg2* mutant than the Δ*Pdpg1* mutant [66]. Since PGs is involved in the degradation of the pectin backbone and pectin is the major component of citrus peel [53,67], these results indicate that plant cell wall degradation enzyme genes *Pdpg1* and *Pdpg2* are involved in *Pd* pathogenicity probably via degradation of pectin from citrus peel [66]. 

Two genes encoding enzymes necessary for fungal cell wall integrity were also reported to regulate *Pd* pathogenicity [68,69,70]. Several chitin synthase genes were found to be upregulated during *Pd* infection of citrus fruit and one chitin synthase mutant Δ*PdChsVII* was defective in *Pd* cell wall integrity and virulence on citrus fruit [68,69]. Since chitin is an essential component of the fungal cell wall but is not present in plants [71], chitin can be considered an important target for developing new antifungal drugs for controlling citrus postharvest green mold. Protein O-mannosyltransferases (Pmts) catalyze the first step in protein O-mannosylation, which is essential for protein modification and is involved in cell wall synthesis [72]. A gene *Pdpmt2* encoding O-mannosyltransferase was proven to regulate *Pd* cell wall integrity and pathogenicity because the Δ*Pdpmt2* mutant was defective in cell wall integrity and disease symptoms on citrus fruit [70]. These results highlight the involvement of fungal cell wall integrity in *Pd* pathogenicity.

### 3.3. Protein Kinases in Pd Pathogenicity

Protein kinases (PKs) catalyze the reversible phosphorylation of proteins and the role of PKs in fungal pathogenicity has been investigated during the last two decades [73]. Mitogen-activated protein kinase (MAPK) and cyclic adenosine monophosphate (cAMP)-protein kinase A(PKA) are two conserved and well-studied PK cascades proven to regulate fungal pathogenicity [73]. MAPK-mediated signaling pathways are highly conserved in all eukaryotic organisms, and filamentous fungi have three MAPKs orthologous including Hog1, Slt2, and Fus3/Kss1MAPKs [74]. The Hog1, Slt2, and MAPKs orthologous in *Pd*, termed Pdos2, PdSlt2, and PdMpkB, were identified and their roles in *Pd* pathogenicity were investigated [75,76,77]. Compared with the wild type strain, all Δ*Pdos2*, Δ*PdSlt2*, and Δ*PdMpkB* mutants showed reduced virulence on citrus fruit and even the Δ*PdMpkB* mutant failed to induce green mold decay on citrus fruit [75,76,77]. Although all the three protein kinases are involved in *Pd* pathogenicity, they probably function in different ways. Cell wall integrity of *Pd* is defective in the Δ*Pdos2* mutant [77], which indicates that Pdos2 is involved in *Pd* pathogenicity possibly by maintaining cell wall integrity. Transporter genes (*PdMfs1-6, PMR1, and PMR5*) and sterol demethylase genes (*PdCYP51A* and *PdCYP51B*) are upregulated and downregulated, respectively, in the Δ*PdSlt2* mutant [76], which is similar as Δ*PdSte12* mutant (see Section 3.1). Thus, PdSlt2 is involved in *Pd* pathogenicity probably via negative regulation of transporters and positive regulation of sterol demethylases. Several genes encoding cell wall-degrading enzymes, such as cutinase, pectin lyase, and rhamnogalacturonan acetylesterase are significantly downregulated in the Δ*PdMpkB* mutant [75], suggesting that PdMpkB is involved in *Pd* pathogenicity probably via positive regulation of cell wall-degrading enzymes.

In addition to these conserved PK signaling networks, new PKs related to fungal pathogenicity were identified in recent years [73]. The role of sucrose nonfermenting protein (SNF1p), which belongs to serine/threonine protein kinases and controls carbon source use in microorganisms, in *Pd* pathogenicity was reported [78]. The Δ*PdSNF1* mutant showed reduced disease symptoms and the expression levels of several cell wall-degrading enzyme genes, including pectin lyase 1(*PNL1*), xylanase 1 (*XY1*), pectate lyase 1 (*PL1*), and exo-polygalacturonase 2 (*EXPG2*), are upregulated in the wild type strain but not in the Δ*PdSNF1* mutant [78], which indicates that PdSNF1 is involved in *Pd* pathogenicity probably via positive regulation of cell wall-degrading enzymes.

### 3.4. Transporters in Pd Pathogenicity

Fungal transporters, mainly including ATP-binding cassette (ABC) transporters and the major facilitator superfamily (MFS) transporters, are known for their involvement in fungicide resistance by promoting the efflux of toxic compounds [79]. Two ABC transporters (PMR1 and PMR5) and two MFS transporters (PdMfs1 and PdMfs2) were proven to regulate *Pd* fungicide resistance [80,81,82,83,84]. PdMfs1 and PdMfs2 are also involved in *Pd* pathogenicity because both Δ*Pdmfs1* and Δ*Pdmfs2* mutants are defective in *Pd* virulence on citrus fruit compared with the wild type strain [80,81]. Other MFS transporters, including Cfp in *Cercospora kikuchii*, Ctb4 in *C. nicotianae*, and ToxA in *C. carbonum*, were also proven to regulate fungal pathogenicity by secreting toxins because disruption of these MFS genes greatly reduces the accumulation of related toxins and results in attenuated virulence [85,86,87]. Whether PdMfs1 and PdMfs2 also regulate *Pd* pathogenicity by secreting toxins, such as tryptoquialanine A and tryptoquialanine C [38], remains to be investigated. 

In addition to the two major facilitator superfamily transporters PdMfs1 and PdMfs2, a putative sucrose transporter PdSUT1 is involved in *Pd* pathogenicity, which is proven by the Δ*PdSUT1* mutant showing reduced *Pd* virulence on citrus fruit compared with the wild type strain [88]. Since fungal sucrose transporter is a homologue to plant sucrose transporters [89], and sucrose is induced in citrus fruit upon *Pd* infection [90], we infer that PdSUT1 is involved in *Pd* pathogenicity possibly by transporting sucrose from host citrus fruit to promote *Pd* development. 

### 3.5. Other Genes in Pd Pathogenicity

Glucosylceramides (GlcCers) are important compositions of membrane lipids in fungi and a gene encoding GlcCer synthase, *PdGcs1*, was proven to regulate *Pd* pathogenicity [91]. The complete loss of production of GlcCers (d18:1/18:0 and d18:2/18:0 h) and a decrease in fungal growth and virulence on citrus fruit were observed in the Δ*PdGcs1* mutant compared with the wild type strain [91], indicating that PdGcs1 is involved in *Pd* pathogenicity probably by controlling the biosynthesis of GlcCers in fungal membrane compositions. Since GlcCers are conserved pathogenicity factors in different fungal pathogens and the structure of fungal GlcCers are remarkably distinct from their counterparts in animal cells, fungal GlcCers are ideal targets for new drugs to control fungal diseases [92]. The camelid single domain antibodies (VHHs) generated against fungal GlcCers were reported to inhibit the growth of *B. cinerea* in vitro and the final disease symptom on tomato leaves [93]. Whether VHHs can also inhibit *Pd* growth and virulence to finally control citrus postharvest green mold remains to be investigated.

Adenylyl cyclase converts ATP to form cAMP in the conserved cAMP-PKA signaling cascade, [94], and an adenylyl cyclase gene *Pdac1* was identified in *Pd* [95]. Pdac1 is required for cAMP production and *Pd* pathogenicity because the Δ*Pdac1* mutant showed decreased accumulation of cAMP and reduced virulence on citrus fruit compared with the wild type strain [95]. These results highlight the involvement of cAMP-mediated signaling in the pathogenicity of postharvest pathogens. 

Fungal CYP51s belong to the cytochrome P450 monooxygenase (CYP) superfamily and are essential for ergosterol synthesis in fungal membranes [96]. Fungal CYP51s, such as PdCYP51A and PdCYP51B from *Pd*, are known for their involvement in resistance to fungicides inhibiting ergosterol synthesis, but increasing evidence shows that some of them also play a role in fungal growth and virulence [96]. A recent study showed that overexpression of *PdCYP51B* increased *Pd* virulence on citrus fruit compared with the wild type strain, indicating that PdCYP51B contributes to *Pd* pathogenicity possibly by regulating ergosterol synthesis in *Pd* membranes [97]. Noticeably, *PdCYP51B* was reported to be positively modulated by other *Pd* pathogenicity factors including transcription factors PdSte12 [48], PdsreA [61], and PdsreB [62], and protein kinase PdSlt2 [76], suggesting that PdCYP51B may be a conserved downstream target in *Pd* pathogenicity.

## 4. Fruit Resistance against *Pd* Infection

**Figure 2:**
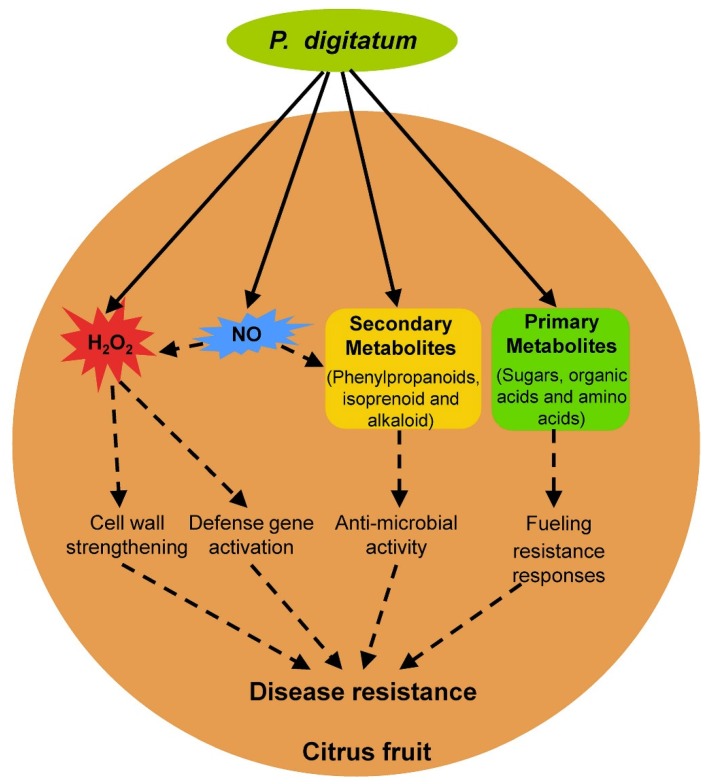
Overview of resistance responses in citrus fruit against *Penicillium digitatum* infection. Solid and dotted arrows indicate direct and tentative stimulatory modification, respectively.

Compared with *Pd* pathogenicity, the understanding of fruit resistance against *Pd* infection has lagged. *Pd* can infect nearly all citrus cultivars and no true resistant citrus cultivars exist that show immunity to postharvest green mold, although aggressiveness of postharvest green mold including lesions and incidence varies significantly in different citrus cultivars [34,98]. Upon *Pd* infection, immature citrus fruit shows a lower lesion diameter than commercial and over-mature harvests citrus fruit [99], which indicates that fruit maturity plays an important role in the development of postharvest green mold similar to other postharvest fungal diseases [2]. In addition to intrinsic fruit maturity, several fruit resistance responses, including reactive oxygen species, nitric oxide, secondary metabolites, and primary metabolites, were identified in the citrus fruit–*Pd* interaction (Figure 2).

### 4.1. Reactive Oxygen Species and Nitric Oxide in Citrus Fruit Resistance

Reactive oxygen species (ROS) and nitric oxide (NO) are two important and closely connected signaling molecules in the plant response to biotic stresses [100]. The production of ROS, mainly including hydrogen peroxide (H_2_O_2_) and superoxide anion (O_2_^−^) via consumption of oxygen in an oxidative burst, plays an important role in the plant response to pathogen infection, usually during the early infection of pathogens [101]. The role of ROS in leaf disease resistance has been well documented and ROS are involved in leaf disease resistance mainly by inducing the hypersensitive response (HR), which is associated with restricted pathogen growth, causing strengthening of host cell walls via cross-linking of glycoproteins, and activating defense related genes [101]. However, little is known about the role of ROS in fruit resistance to postharvest pathogens [2]. An H_2_O_2_ burst occurs in citrus fruit during early *Pd* infection, and exogenous H_2_O_2_ treatment of citrus fruit increases the resistance of citrus fruit against *Pd* infection [4,32]. H_2_O_2_ content and resistance to *Pd* in citrus fruit decline after treatment with the antioxidant melatonin [32]. Further transcriptome analysis showed that cell wall encoding genes and defense related genes are significantly upregulated and downregulated, respectively, upon on melatonin treatment [32]. These results indicate that ROS is involved in citrus fruit resistance against *Pd* infection probably via cell wall strengthening and defense gene activation. 

As a diatomic free radical gas, NO is emerging as a key regulator of diverse plant cellular processes that can interact with other signaling molecules such as H_2_O_2_ in plant resistance [100,102,103]. A nitrosative also occurs, resulting in the synthesis of NO in the plant response to pathogens, usually during the early pathogen infection [104]. Upon exogenous NO treatment, the resistance of citrus fruit against the postharvest fungal pathogen *Colletotrichum gloeosporioides* was enhanced along with increased H_2_O_2_ accumulation [105]. Exogenous NO treatment can also enhance the resistance of other fruits including tomato and peach against postharvest pathogens [106,107,108,109]. Noticeably, exogenous NO improved the resistance of peach fruit to postharvest pathogen *Monilinia fructicola* by activating the phenylpropanoid pathway [106], which plays an important role in plant disease resistance and was reported to regulate citrus fruit resistance against *Pd* infection (see Section 4.2). The role of NO in citrus fruit resistance against *Pd* infection was investigated preliminarily and exogenous NO treatment enhanced postharvest disease resistance in citrus fruit to *Pd* [110]. This increasing evidence highlights the involvement of NO in fruit resistance to postharvest pathogens; the detailed function mechanism of NO in citrus fruit resistance against *Pd* infection remains to be investigated in the future.

### 4.2. Plant Metabolism in Citrus Fruit Resistance

Different from primary metabolites, secondary metabolites produced by plants are primarily involved plant defense against herbivores and microbes [111]. Metabolic analysis showed that flavanones, flavones, polymethoxylated flavones, and scoparone are induced in citrus fruit upon *Pd* infection [112]. Transcriptional expression analysis showed that many phenylpropanoid biosynthetic genes are induced in citrus fruit upon *Pd* infection [112,113]. *Pd* infection also induces the expression of citrus genes involved in other secondary metabolisms, including isoprenoid, alkaloid, caffeine synthase, tropinone reductase, and berberine bridge-like [113]. Phenylpropanoids belong to the largest group of secondary metabolites produced by plants and are involved in plant disease resistance mainly by acting as phytoalexins with anti-microbial activity [114]. Treatment with phenylpropanoids or their derivatives improved the resistance of citrus fruit to *Pd* [115]. These results highlight the involvement of secondary metabolism in citrus fruit resistance to *Pd*. 

Increasing evidence indicates that primary metabolism in ATP generation is also involved in plant disease resistance [116,117]. Energy plays a critical role in the execution of plant defense responses due to the expression of many genes from multiple defense pathways, and the main role of primary metabolites in plant disease resistance is fueling defense responses as an energy provider [116,117]. Many genes involved in primary metabolic pathways, such as the citrate cycle (TCA cycle), glycolysis/gluconeogenesis, and biosynthesis of amino acids, are significantly upregulated in plant resistance to different pathogens [118]. To meet the increased demand for carbon in many defense responses, such as the induction of antimicrobial phenylpropanoids, the plant shunts amino acids into energy-generating pathways such as the TCA cycle [116]. The increase in carbohydrates is observed in plant resistance to several different pathogens [116]; floating with glucose, fructose, and sucrose solutions induced several tobacco defense-related genes [119]. However, little is known about primary metabolites in fruit disease resistance compared with leaf disease resistance. Exogenous ATP treatment maintained higher energy levels of harvested litchi fruit and increased fruit resistance to *Peronophythora itchii* [120], which highlights the role of energy metabolism in fruit response to postharvest pathogen infection. Metabolomic profiling of citrus fruit with enhancement of disease resistance by postharvest heat treatment (HT) showed that HT induced the accumulation of sugars [121]. There was a significant change (upregulated predominantly) in primary metabolites including sugars, organic acids, and amino acids in citrus fruit upon *Pd* infection [90]. These results indicate that primary metabolites are probably also involved in citrus fruit resistance against *Pd* infection by fueling resistance responses. 

### 4.3. Resistance Mediated by Other Fruits

Although considered an important postharvest pathogen, *Pd* was previously described to show a limited host range and infect fruits belonging to the Rutaceae family exclusively [5,122,123]. However, increasing evidence shows that *Pd* is also an opportunistic pathogen of pome fruits (apple and pear) and stone fruits (nectarine and plum) [124,125], which were previously thought to be nonhosts of *Pd* [126]. Fruit ripeness and postharvest storage significantly impact the infection and colonization of *Pd* in these pome and stone fruits [126,127,128]. Upon *Pd* inoculation, immature apple fruit showed no decay symptoms, but a small number of apple fruit from commercial harvest showed a decay limited to the initial infection site and over-mature apple fruit developed a complete rot similar with the well-known host citrus fruit [126]. Lignin content and H_2_O_2_ production were highest in immature apple fruit [126,129], which reveals the involvement of lignification and H_2_O_2_ in apple fruit resistance to *Pd*. Transcriptomic profiling of apple fruit in response to *Pd* infection showed that apple phenylpropanoid metabolism-related genes are significantly upregulated [130], which is similar as transcriptomic profiling of citrus fruit in response to *Pd* infection [112,113]. Proteome analysis and protein carbonyls (oxi-proteome) analysis formed by ROS revealed that the oxidation of proteins related to energy metabolism and the prevention of free nutrient movement are involved in apple resistance against *Pd* infection [131]. 

## 5. Open Questions

### 5.1. What Are the Roles of Virulence-Associated Effectors in Pd Pathogenicity?

About 9000 genes exist in the *Pd* genome [5]; thus, identification and functional analysis of more pathogenicity-related *Pd* genes will further reveal *Pd* pathogenicity at the molecular level. Virulence-associated effectors, which are secreted by pathogens to modulate host defense responses or cell physiology to promote plant susceptibility [132,133], are especially worthy of attention in *Pd*. Since effectors are important pathogenicity factors of plant pathogens and play a key role in plant–pathogen interactions [134], effector biology is a research hotspot, with many pathogenic fungi receiving considerable attention [133]. Effectors can be used to evaluate pathogen evolution and prevalence and be engineered to develop plant disease resistant crops [135]. HIGS of virulence-associated effector *PstGSRE1* in transgenic wheat plants significantly improved wheat resistance to the stripe rust pathogen *Puccinia striiformis* f. sp. *tritici*, one of the most important fungal pathogens in wheat [136]. However, little is currently known about effectors in postharvest fungal pathogens [2]. There are 552 secreted protein encoding genes in the *Pd* genome and some of them encode putative small cysteine-rich proteins (CRPs) such as effectors or homologous to effector proteins from other pathogenic fungi [5]. Some secreted protein encoding genes were proven to be putative pathogenicity-related genes of *Pd* because their expressions are induced during *Pd* infection on citrus fruit [7]. Functional analysis of these putative effectors in *Pd* pathogenicity will fill the research gap about effector function in postharvest fungal pathogens and provide novel insights into fruit–pathogen interactions.

### 5.2. Can the Revolutionary Genome Editing Biotechnology Be Used to Generate Citrus Varieties Resistant to Postharvest Green Mold?

Despite having the citrus genome sequence and citrus genetic transformation system [25,26], no other citrus gene was reported to be required for fruit resistance against *Pd* infection. Many citrus genes were proven to regulate citrus resistance to other diseases using transgenic technology [26], and whether these genes are also involved in citrus resistance to postharvest green mold remains to be investigated. Among plant defense-related genes, disease susceptibility genes (S genes), which are required for plant susceptibility to diseases and usually function as negative regulators of plant defense and targets of pathogen effectors [137], have received increased attention because disabling plant S genes to achieve durable and broad-spectrum resistance is a novel breeding strategy [138]. The revolutionary genome editing biotechnology has broad application prospects in plant breeding of disease resistant crops because it allows plant breeding without introducing a transgene and can produce novel plants that are similar or identical to plants generated by conventional breeding techniques [17,139]. Plant S genes represent good targets for genome editing to create disease resistant crops; several transgene-free and disease resistant crops including tomato, cucumber, grape, and apple have been created by genome editing of plant S genes including *MLO*, *eIF4E*, and *DIPM* via most recently, clustered regularly interspaced short palindromic repeats (CRISPR)/CRISPR-associated protein 9 (Cas9) system [140]. The CRISPR/Cas9 gene editing system has been established in citrus, and genome editing of the citrus S gene *CsLOB1* in citrus confers resistance to citrus canker [29,141]. Thus, identification of citrus S genes to postharvest green mold and creating resistant citrus varieties by genome editing of these citrus S genes provide a promising pathway to control citrus postharvest green mold.

## 6. Conclusions

Postharvest fungal diseases on fruit have received increased attention from researchers in different fields mainly including horticulture, plant protection, and food science. Synthetic fungicides, which have noticeable health or environmental risks, are still the main method used to control them in current fruit storage. Although many nonchemical treatments, mainly including biocontrol agents, natural compounds, UV, hot water treatment, and salts have been used for controlling postharvest fungal diseases on fruit [142,143], these diseases still lead to huge economic losses worldwide every year. Increasing evidence reveals that investigating molecular mechanisms of plant–pathogen interactions is essential for developing novel and safer strategies for durably controlling plant diseases. Thus, this review focused on recent advances in the citrus fruit–*Pd* interaction, providing significant insights into fruit–pathogen interactions and is beneficial for developing novel and safer strategies for controlling citrus postharvest green mold. A total of 19 *Pd* genes mainly encoding transcription factors, cell wall-related enzymes, protein kinases, and transporters are required for *Pd* pathogenicity. Of these *Pd* pathogenicity factors, PdChsVII and PdGcs1 are ideal targets for new drugs to control citrus postharvest green mold. Whether HIGS or SIGS of *Pd* pathogenicity factors can be used to control citrus postharvest green mold remains to be investigated. In addition, several fruit resistance responses, including ROS, NO, secondary metabolites, and primary metabolites, are involved in citrus fruit resistance against *Pd* interaction. No other citrus gene has yet been reported to regulate citrus fruit resistance. Identification and functional analysis of citrus genes that regulate citrus fruit resistance against *Pd* infection will be conducted using transgenesis or genome editing.
